# A Genetic Model Reveals Biological Features of Neonatal CD4 Helper Cells Undergone Homeostasis in Mice

**DOI:** 10.3389/fcell.2021.659744

**Published:** 2021-03-11

**Authors:** Lei Lei, Xingzhe Zhang, Xiaofeng Yang, Yanhong Su, Haiyan Liu, Hang Yang, Jinli Wang, Yujing Zou, Xin Wang, Anjun Jiao, Cangang Zhang, Huiqiang Zheng, Jiahui Zhang, Dan Zhang, Lin Shi, Xiaobo Zhou, Chenming Sun, Baojun Zhang

**Affiliations:** ^1^Department of Pathogenic Microbiology and Immunology, School of Basic Medical Sciences, Xi’an Jiaotong University, Xi’an, China; ^2^Department of Kidney Transplantation, Nephropathy Hospital, The First Affiliated Hospital of Xi’an Jiaotong University, Xi’an, China; ^3^Institute of Infection and Immunity, Translational Medicine Institute, Xi’an Jiaotong University Health Science Center, Xi’an, China; ^4^Key Laboratory of Environment and Genes Related to Diseases, Ministry of Education, Xi’an Jiaotong University, Xi’an, China; ^5^Xi’an Key Laboratory of Immune Related Diseases, Xi’an, China; ^6^Duke University Medical Center, Durham, NC, United States

**Keywords:** neonate, adulthood, tracked T cells, homeostasis, differentiation

## Abstract

CD4^+^ T cells are essential for regulating effective immune response to pathogens and immune balance. Recent studies have demonstrated the unique features of T cells in neonate mice, such as more sensitive to antigen response and preference toward T helper 2 (Th2) response and regulatory T cells (Tregs) differentiation. However, the biological characteristics of neonatal age-derived CD4^+^ T cells following homeostasis remain unclear. Here we utilized a lineage tracing model of *TCR*δ*^*CreER*^R26^*ZsGreen*^* to mark neonatal- and adult-derived CD4^+^ T cells followed by a combination analysis of activation, proliferation, survival, and differentiation. Our results showed that neonatal CD4^+^ T cells had higher capacity of activation, proliferation, apoptosis, and differentiation toward Th2 and T helper 17 (Th17) lineages, accompanied by a reduced potential for T helper 1 (Th1), T helper 9 (Th9), and Treg lineages. In contrast, tracked neonatal CD4^+^ T cells exhibited similar characters of above-mentioned of tracked adult cells in adult mice. Therefore, our data support a natural requirement for CD4^+^ T cells to acquire fully-equipped functional potentials of adult cells.

## Introduction

In adults, CD4^+^ T helper cells play a pivotal role in adaptive immunity including activation of innate cells, helping B cells to produce antibodies, and generation of CD8^+^ cytotoxic T cells (Luckheeram et al., 2012). The helper function and population size are determined by their biological characters such as activation, proliferation, and survival. However, a dysregulated response of CD4^+^ T cells can lead to the occurrence of various autoimmune diseases (Pawlak et al., 2020). Under specific cytokine conditions and activation of intracellular signals, naïve CD4^+^ T cells are capable to differentiate into different subsets of Th1, Th2, Th9, and Th17 cells, producing inflammatory cytokines such as interferon-gamma (IFN-γ), interleukin-4 (IL-4), interleukin-9 (IL-9), and interleukin-17 (IL-17) to mediate immune responses (Ruterbusch et al., 2020; Yan and Richmond, 2020). Naïve CD4^+^ T cells are also able to develop into natural Treg cells (nTreg) in the thymus and differentiate into induced regulatory T cells (iTreg) in the presence of T cell receptor (TCR) and transforming growth factor beta (TGFβ) stimulation to maintain immune balance, preventing the occurrence of autoimmune diseases (Caza and Landas, 2015).

However, neonatal CD4^+^ T cells exhibit distinct features and mediate different immune response from adult cells. Neonatal CD4^+^ T cells are more sensitive to TCR stimulation (Wilson and Kollmann, 2008). In humans, there is a higher incidence of acute graft-versus-host disease (GVHD) in neonates than adults, which can be attributed to the activation potential of CD4^+^ T cells (Rocha et al., 2000; Ballen and Spitzer, 2011). Mechanistically, the distinct transcriptome or epigenetic landscape allows neonate T cells to respond more strongly self-antigens or foreign antigens (Palin et al., 2013; Rudd, 2020).

In neonates, CD4^+^ T cells have been characterized as relatively immature, associated with limited immune protection (Nelson et al., 2015). Human neonatal CD4^+^ recent thymic emigrants (RTEs) demonstrated a defective potential to differentiate into IFN-γ secreting Th1 cells (Haines et al., 2009), which is in line with the reduced expression of Th1 cytokines *in vitro* (Chen et al., 2006). In contrast, neonatal CD4^+^ T cells differentiate into Th2 cells more readily than adult CD4^+^ T cells. This can be attributed to hypo-methylation of Th2 cytokine gene loci in neonates compared to adults (Rose et al., 2007; Debock and Flamand, 2014). Neonatal CD4^+^ T cells from human cord blood have limited potential to differentiate into Th17 cells given stimulation with interleukin-1 beta (IL-1β), interleukin-6 (IL-6), and interleukin-23 (IL-23) in comparison to adult peripheral blood mononuclear cells (PBMCs), which is mainly caused by low level of RORC2 transcription (Hofstetter et al., 2007; de Roock et al., 2013). In the mouse model of experimental autoimmune encephalomyelitis (EAE), neonatal mice also showed a lower level of IL-17-producing cells compared to adult mice (Hofstetter et al., 2007; de Roock et al., 2013). However, neonatal CD4^+^ T cells preferentially differentiate into Treg cells compared adult CD4^+^ T cells under the stimulation of anti-CD3 and anti-CD28 antibodies with or without TGFβ *in vitro* (Fernandez et al., 2008; Wang et al., 2010). Overall, the immune competency in neonates is relatively dormant.

The distinct immunological characteristics of neonatal and adult CD4^+^ T cells indicate that neonatal cells undergo a maturation step during homeostasis. Recent study found that adult CD8^+^ T cells generated at the neonatal stage preferentially become memory-like cells under unchallenged conditions, and differentiate into effectors following infection (Smith et al., 2018). However, little is known about the immunological features of adult CD4^+^ T cells generated at the neonatal age. Here, we utilized a recently developed lineage tracing model to examine the phenotypical and functional differences among neonatal, adult, tracked neonatal (adult cells generated at neonatal age) and tracked adult (adult cells generated at adult age) CD4^+^ T cells. We found a higher percentage of effector memory T cells (TEM, CD44^hi^CD62L^–^) and center memory T cells (TCM, CD44^hi^CD62^+^) in lymph nodes (LNs) but not in spleens of neonatal mice compared with adult mice, as well as an increase of TEM and TCM cells proportions in tracked-neonatal cells. Neonatal CD4^+^ T cells were sensitive to TCR activation, proliferation, and activation-induced cell death, whereas tracked-neonatal cells behaved similarly as adult and tracked-adult cells. Finally, neonatal CD4^+^ T cells more readily differentiated into Th2, Th17, and Treg cells rather than Th1 cells. In contrast, tracked-neonatal CD4^+^ T cells exhibited similarly differentiation potential into all Th lineages examined. Collectively, our data demonstrated that neonatal CD4^+^ T cells acquired the phenotypical and functional characteristics of adult cells after homeostatic process.

## Materials and Methods

### Mice and Reagents

*TCR*δ*^*CreER*^R26^*ZsGreen*^* mice were developed and used as described previously (Zhang et al., 2015). The transgenic mouse model can successfully track T cells generated from one wave of developing thymocytes by a lineage-specific and inducible Cre-controlled reporter. All mice were bred and maintained in the specific pathogen-free conditions by Xi’an Jiaotong University Division of Laboratory Animal Research. All the procedures were approved by the Institutional Animal Care and Use Committee of Xi’an Jiaotong University.

The antibodies used are as follows: APC/Cy7 anti-mouse CD4 (GK1.5), PE/Cy7 anti-mouse/human CD44 (IM7), APC anti-mouse CD62L (MEL-14), PE anti-mouse CD69 (H1.2F3), PE anti-mouse CD25 (PC61), PE/Cy5 anti-mouse CD25 (PC61), Pacific Blue^TM^ anti-mouse Ki-67 (16A8), PE Annexin V (Cat # 640947), Pacific Blue^TM^ anti-mouse FOXP3 (MF-14), Pacific Blue^TM^ anti-mouse IFNγ (XMG1.2), Brilliant Violet 421 anti-mouse IL-4 (11B11), PE anti-mouse IL-9 (RM9A4), Alexa FluorR 647 anti-mouse IL-17A (TC11-18H10.1), Purified anti-mouse CD3 (145-2C11), purified anti-mouse CD28 (37.51). All antibodies were purchased from BioLegend (San Diego, CA, United States). 7AAD Viability Staining solution (Cat # 420404), Fixation Buffer (Cat # 420801), and Intracellular Staining Perm Wash Buffer (Cat # 421002). Transcription Factor Fixation/Permeabilization Concentrate and Diluent were purchased from eBioscience.

### Tamoxifen Treatment

Newborn *TCR*δ*^*CreER*^R26^*ZsGreen*^* pups were given five drops of tamoxifen (10 mg/ml) daily for 5 days. Six-week-old *TCR*δ*^*CreER*^R26^*ZsGreen*^* mice were treated with three doses of 1 mg tamoxifen intraperitoneally every other day.

### Flow Cytometry

Single cells were obtained from spleen and lymph-node of indicated mice. For cell surface analysis, 1∼5 × 10^6^ cells per sample were stained with Abs in the dark at 4°C for 30 min. After washing with cold FACS buffer (1 × PBS supplemented with 2% FBS), cells were analyzed using CytoFLEX flow cytometer (BECKMAN COULTER). Flowjo software (CytExpert) was used for data recorded and analyzed.

To analyze intracellular transcriptional factors, after a 30-min surface staining, cells were fixed and permeabilized according to the manual of Foxp3 kit, followed by anti-foxp3 antibody staining and FACS analysis.

For cytokine analysis, cell samples were stimulated *in vitro* with PMA/Ionomycin in the presence of Brefeldin A (BioLegend) and Monensin (BioLegend) for 4 h. Cells were washed and stained with anti-CD4 Abs. After a 30-min incubation and wash, cells were fixed and permeabilized using Fixation/Permeabilization buffer (BioLegend), and stained with IFN-γ, IL-4, and IL-9 antibodies and FACS analysis.

### T Cell Culture

Lymphocytes were cultured in RPMI 1640 medium (GIBCO) supplemented with 100 U/mL of penicillin, 100 μg/mL of streptomycin, 0.05 mM of β-mercaptoethanol, and 10% fetal bovine serum (GIBCO) with 2.5 μg/ml anti-CD3 and 1 μg/ml anti-CD28 Abs for indicated hours. For proliferation and apoptosis *in vitro*, cell samples were stained with indicated surface markers. After wash with FACS buffer, intracellular staining with anti-Ki67 labeled proliferating cells, or apoptosis was analyzed with 7AAD/Annexin V using apoptosis detection kit (1 × binding buffer).

### T Helper Cell Differentiation *in vitro*

Naive T cells (CD4^+^CD25^–^CD44^–^CD62L^+^) were sorted by BD FACSAria^TM^ II cell sorter (BD Biosciences, San Jose, CA, United States) from spleen and LNs. Purified cells were cultured in 96-well plates coated with 2.5 μg/mL anti-CD3, and 0.5 μg/mL anti-CD28 Abs in the presence of different cytokine cocktails: Th1: IL-2 (50 U/mL), IL-12 (20 ng/mL), and anti-IL-4 Abs (10 μg/mL); Th2: IL-2 (50 U/mL), IL-4 (100 ng/mL), anti-IFN-γ Abs (10 μg/mL), and anti-IL-12 Abs (10 μg/mL); Th9: IL-4 (20 ng/mL), anti-IFN-γ Abs (10 μg/mL), and TGF-β1 (2 ng/mL); Th17: IL-6 (50 ng/mL), IL-23 (50 ng/mL), IL-1β (10 ng/mL), TGF-β1 (5 ng/mL), anti-IL-4-Abs (10 μg/mL), and anti-IFN-γ-Abs (10 μg/mL); iTreg: TGF-β1 (5 ng/mL), IL-2 (50 U/mL), anti-IL-4 Abs (10 μg/mL), and anti-IFN-γ Abs (10 μg/mL). 96 h post culture, cells were performed antibody staining and FACS analysis.

### Statistical Analysis

Statistical analysis was applied to technical replicates for each experiment. Each experiment was independently repeated three times. Two-tailed Student’s *t* test was used for all statistical calculations using GraphPad Prism 7 software. The level of significance is indicated as ^∗^*P* < 0.05, ^∗∗^*P* < 0.01, ^∗∗∗^*P* < 0.001, and ^****^P < 0.0001.

## Results

### Effector/Memory-Like Cells Accumulate in Tracked-Neonatal CD4^+^ T Cells

**FIGURE 1:**
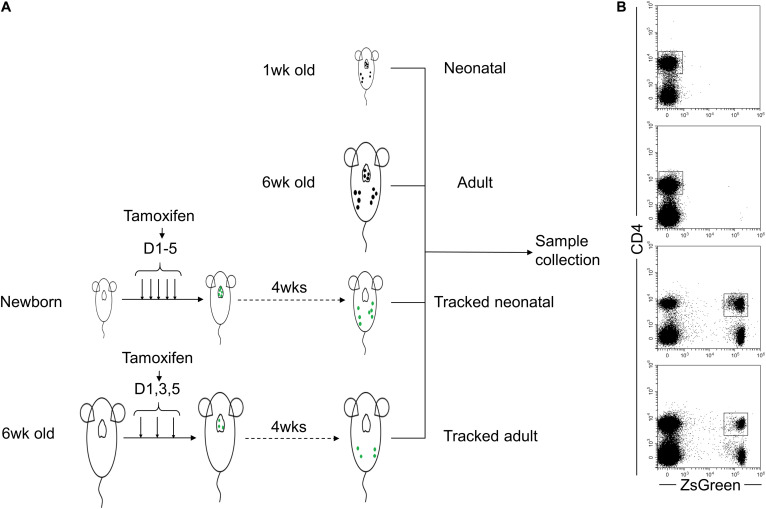
Schematic of tracked CD4^+^ T cells generated at different stages using ZsGreen reporter**. (A)** The four groups of cells are included as follows. Neonatal and adult CD4^+^ T cells directly isolated from spleens and lymph nodes of 1-week-old and 6-week-old wt C57BL/6 mice were Group1 (Neonatal, N) and Group2 (Adult, A), respectively. Neonatal mice were treated with five doses of tamoxifen within 5 days after birth. Four weeks later, ZsGreen^+^CD4^+^ cells isolated from spleens and lymph nodes were Group 3 named as Tracked-neonatal (TN). Young adult mice (6- week-old mice) were treated with three doses of tamoxifen every other day. 4 weeks post-treatment, ZsGreen^+^CD4^+^ T cells collected from spleens and lymph nodes were Group 4, named as Tracked adult (TA). **(B)** Representative FACS plots of ZsGreen-tracked and non-tracked CD4^+^ T cells from four groups in panel **(A)**.

In order to investigate the effect of homeostatic process on biological characteristics of neonatal CD4^+^ T cells *in vivo*, we utilized the *TCR*δ*^*CreER*^R26^*ZsGreen*^* mouse model described in the previous study (Zhang et al., 2016) to track CD4^+^ T cells generated at the neonatal age in adult mice. As shown in Figure1A, new-born and 6-week-old *TCR*δ*^*CreER*^R26^*ZsGreen*^* mice were treated with tamoxifen. Four weeks later, we collected spleens and LNs from tracked-neonatal mice, tracked-adult mice, and control mice (untreated 1-week-old neonatal mice and 6-week-old adult mice). Clearly, a ZsGreen-positive population of CD4^+^ T cells was observed (Figure 1B).

**FIGURE 2:**
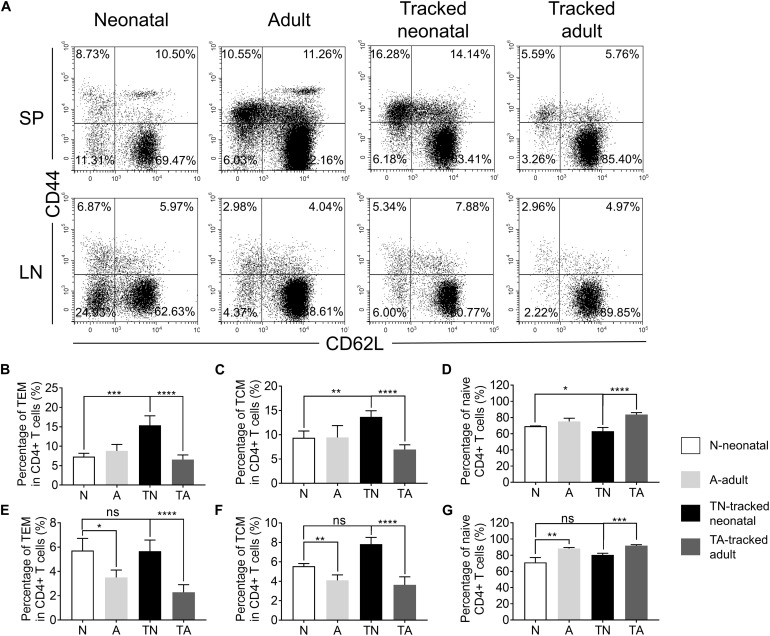
The presence of naive and effector/memory-like populations in tracked and non-tracked CD4^+^ T cells. **(A)** Representative FACS plots of Naïve, effector memory (TEM) and central memory (TCM) cells in four groups. The statistical percentages of TEM cells in CD4^+^ splenocytes **(B)** and lymph nodes **(E)**. The statistical percentages of TCM cells in CD4^+^ splenocytes **(C)** and lymph nodes **(F)**. The statistical percentages of naive T cells in CD4^+^ splenocytes **(D)** and lymph nodes **(G)**. **(A–G)** *n* = 3 mice; The results shown are representative of three independent experiments. *P < 0.05, **P < 0.01, ***P < 0.001, and ****P < 0.0001.

CD4^+^ T cells, generated from thymus, migrate to spleen and LNs in the periphery. A portion of these cells differentiated into TEM or TCM in the lymphopenic environment (Sallusto et al., 2004). The proportions of TEM and TCM cells in neonatal mice compared to that of adults were similar in spleen (Figures 2A–C) but higher in LNs (Figures 2A,E,F), while the percentage of naïve cells in neonatal mice compared to that of adults were similar in spleen and lower in LNs (Figures 2A,D,G). Similarly, tracked-neonatal mice showed a significant higher percentage of TEM and TCM cells (Figures 2A–C,E,F) and a lower percentage of naïve cells in compared to the other groups (Figures 2A,D,G). Therefore, neonatal CD4^+^ T cells are capable to differentiate into effector cells in the lymphopenic environment throughout the span from neonates to adults.

### Early and Late Activation of Tracked Neonatal CD4^+^ T Cells and Controls

**FIGURE 3:**
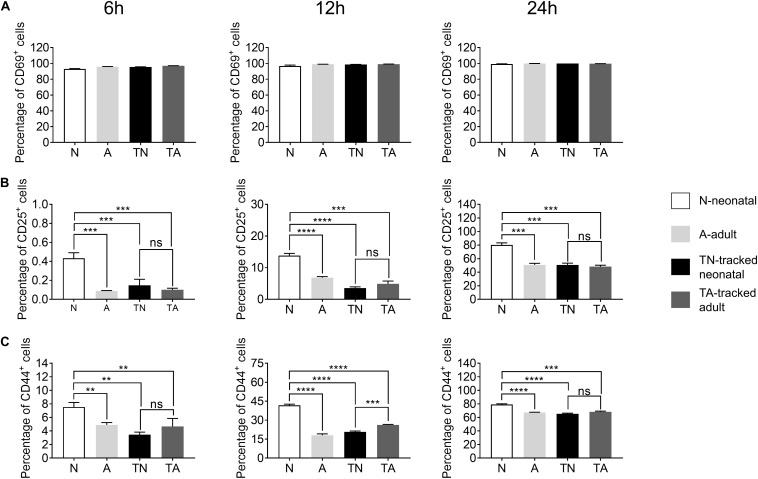
Expression of activation markers in stimulated CD4^+^ T cells. Naïve CD4^+^ T cells from tracked and non-tracked groups were sorted and cultured *in vitro* with 2.5 μg/ml anti-CD3 Ab and 1 μg/ml anti-CD28 Ab for 6, 12, and 24 h. Cells were harvested at indicated times and analyzed by FACS. The percentages of CD69^+^ cells **(A)**, CD25^+^ cells **(B)**, and CD44^+^ cells **(C)** in CD4^+^ T cells after stimulation for 6, 12, and 24 h. The blank column represents neonatal CD4^+^ T cells. The light gray column represents adult CD4^+^ T cells. The black column represents tracked-neonatal CD4^+^ T cells. The dark gray column represents tracked-adult CD4^+^ T cells. **(A–C)** Results shown are representative of three independent experiments. **P < 0.01, ***P < 0.001, and ****P < 0.0001.

Activation is the first step for CD4^+^ T cells to respond to antigens presented by antigen presenting cells (APCs) to initiate helper function during an immune response. We found a similar percentage of CD69^+^ cells among neonatal and adult CD4^+^ T cells after *in vitro* stimulation with anti-CD3 and anti-CD28 Abs for 6–24 h (Figure 3A). In contrast, neonatal CD4^+^ T cells exhibited a higher percentage of CD25^+^ and CD44^+^ proportions compared with adult cells during the course of stimulation (Figures 3B,C). Interestingly, tracked neonatal CD4^+^ T cells showed similar expression of CD25^+^ and CD44^+^ to tracked adult cells (Figures 3B,C). The data suggest that neonatal CD4^+^ T cells are sensitive to TCR stimulation and activation, and demonstrate similar potential to adult cells following the homeostatic process.

### Tracked Neonatal CD4^+^ T Cells Acquire Equal Proliferative Ability as Adult Counterparts

**FIGURE 4:**
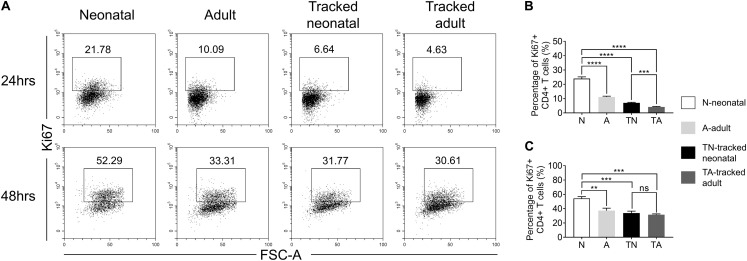
Proliferation of tracked and untracked naïve CD4^+^ T cells after TCR stimulation. Naïve CD4^+^ T cells from tracked and non-tracked groups were sorted and stimulated with 2.5 μg/ml anti-CD3 Ab and 1 μg/ml anti-CD28 Ab *in vitro* for 24 and 48 h. **(A)** Representative FACS plots of Ki67 staining in CD4^+^ T cells at 24 h (the upper row) and 48 h (the bottom row) following stimulation. The statistical percentages of Ki67^+^ cells in tracked and non-tracked CD4^+^ T cells 24 h **(B)** and 48 h **(C)** post stimulation. **(A–C)** Results are representative of three independent experiments. Student *t* test and Mann–Whitney *U* test were used for statistical analysis. **P < 0.01, ***P < 0.001, and ****P < 0.0001.

In response to TCR stimulation, T cells undergo proliferation and expansion. We examined the proliferation ability of naïve CD4^+^ T cells from indicated groups upon stimulation with plate bound anti-CD3 and anti-CD28 Abs. The percentage of Ki67^+^ cells was significantly higher in the neonatal group compared to that in adult group at both 24 h (Figures 4A,B) and 48 h (Figures 4A,C). In contrast, tracked neonatal CD4^+^ T cells showed similar percentage of Ki67^+^ cells compared to adult and tracked-adult cells at both time points (Figure 4). The data demonstrate that higher proliferation potential is a unique feature of neonatal CD4^+^ T cells.

### Activation Induced Apoptosis of Neonatal CD4^+^ T Cells and Controls

**FIGURE 5:**
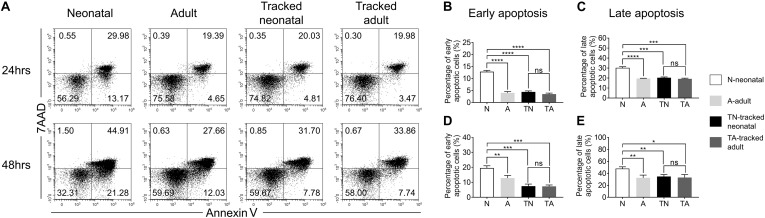
Apoptosis in tracked and untracked CD4^+^ T cells after TCR stimulation. Tracked and non-tracked naïve CD4^+^ T cells were sorted and stimulated with anti-CD3 and anti-CD28 Abs *in vitro* for 24 and 48 h. **(A)** Representative FACS plots of 7AAD and Annexin V staining in CD4^+^ T cells 24 h (the upper row) and 48 h (the bottom row) post-stimulation. The statistical percentages of early apoptotic CD4^+^ T cells (Annexin V^+^ 7AAD^–^) 24 h **(B)** and 48 h **(D)** post-stimulation. **(C)** The percentages of late apoptotic CD4^+^ T cells (Annexin V^+^ 7AAD^+^) 24 h **(C)** and 48 h **(E)** post stimulation. **(A–E)** Results are representative of three independent experiments. Student’s *t* test and Mann–Whitney *U* test were used for statistical analysis. *P < 0.05, **P < 0.01, ***P < 0.001, and ****P < 0.0001.

Following TCR stimulation, a portion of T cells undergo activation-induced cell death. We evaluated CD4^+^ T cell apoptosis after stimulation with anti-CD3 and anti-CD28 Abs for 24 and 48 h using 7AAD and Annexin V staining (Figure 5A). Neonatal CD4^+^ T cells showed significantly higher percentage of early (Annexin V^+^7AAD^–^) and late apoptotic cells (Annexin V^+^7AAD^+^) compared to adult cells at both 24 h (Figures 5B,C) and 48 h (Figures 5D,E). Whereas tracked neonatal CD4^+^ T cells showed similar percentages of both early and late apoptotic cells to adult and tracked adult cells. The data indicate tracked neonatal CD4^+^ T cells acquire superior survival capacity than neonatal cells.

### Differentiation Potential of Tracked Neonatal and Control CD4^+^ T Cells

**FIGURE 6:**
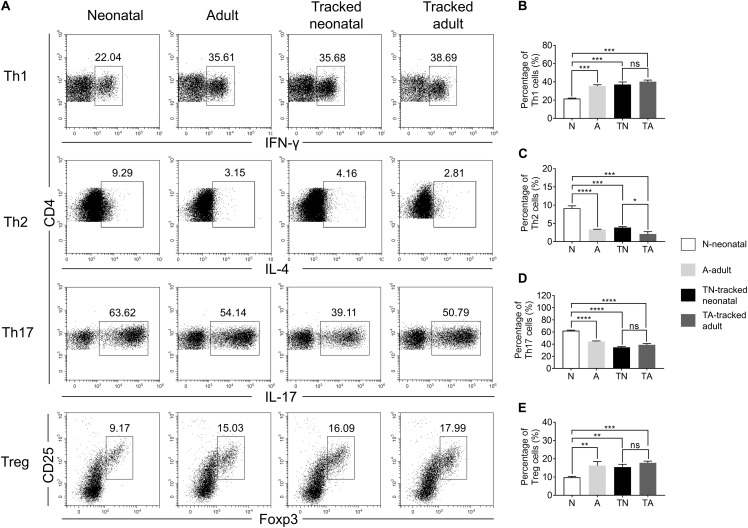
Th cell differentiation of tracked and untracked cells under polarized conditions. Naïve CD4^+^ T cells were sorted from indicated groups of mice and cultured under polarized conditions for 4 days. Intercellular cytokine expression was analyzed by FACS. **(A)** Representative FACS plots of IFNγ, IL-4, IL-17, and Foxp3 staining in CD4^+^ T cells. **(B)** The percentage of IFNγ^+^ cells in CD4^+^ T cells (Th1). **(C)** The percentage of IL-4^+^ cells in CD4^+^ T cells (Th2). **(D)** The percentage of IL-17^+^ cells in CD4^+^ T cells (Th17). **(E)** The percentage of CD25^+^ and Foxp3^+^cells in CD4^+^ T cells (Treg). **(A–E)** Representative results of three independent experiments. Student’s *t* test and Mann–Whitney *U* test were used for statistical analysis. *P < 0.05, **P < 0.01, ***P < 0.001, and ****P < 0.0001.

The functions of CD4^+^ T helper cells depend on their ability during differentiation into specific effector subsets, such as Th1, Th2, Th17, and Treg cells (Luckheeram et al., 2012). We assessed the differentiation potential of effector cells from different groups of naïve CD4^+^ T cells under polarized culture conditions (Figure 6). We showed that neonatal naïve CD4^+^ T cells preferentially differentiated into Th2 (Figures 6A,C) and Th17 (Figures 6A,D) cells but less so for Th1 cells (Figures 6A,B) and Tregs (Figures 6A,E) compared with adult naïve cells. However, tracked neonatal naïve CD4^+^ T cells showed similar differentiation potential for all effector cell types examined compared to tracked-adult cells. Therefore, there are distinct differentiation preferences in neonatal and adult cells, as well as tracked and non-tracked cells.

## Discussion

Accumulating data demonstrate that neonatal CD4^+^ T cells are functionally distinct from that of adults, which directly contributes to the susceptibility to pathogens challenge and allergy development (Debock and Flamand, 2014). However, CD4^+^ T cells in adults are a heterogeneous population generated from both neonatal and adult age. Less is known about the phenotypical and functional features of adult CD4^+^ T cells generated at the neonatal stage following entry into the periphery. Here, we used a well-established lineage-tracing model to track neonatal CD4^+^ T cells to characterize their phenotypes under the steady state condition, as well as the potential of activation, survival, and T helper cell differentiation upon TCR stimulation.

During the homeostatic process under the lymphopenic and stabilized condition, a portion of T cells upregulate CD44 and downregulate CD62L, becoming effector/memory-like cells in peripheral lymph organs (Pepper and Jenkins, 2011). The percentage of CD4^+^CD44^+^ T cells in LNs is significantly higher in neonates than that in adults, while splenic counterparts keep similar between two groups, which indicates that neonatal CD4^+^ T cells undergo stronger expansion in lymphopenic LNs while the size of expansion in spleen is comparable to that of adult cells. Tracked neonatal CD4^+^ T cells also express higher level of CD44 compared to adult and tracked-adult cells, which is consistent with previous findings on CD8^+^ T cells generated at the neonatal stage (Smith et al., 2018). It is possible that tracked neonatal CD44^+^ cells are derived from the retention of neonatal counterparts since tracked adult cells did not show a higher percentage of CD44^+^ cells compared to adult cells during the 4-week window. We have previously shown that there is an increase of CD44^+^ cells during a tracking window of 8 week or 1.5 year (Zhang et al., 2016). Therefore, the length of homeostatic process will determine the proportion of effector/memory-like cells.

Neonates exhibit a compromised immune defense against pathogen infection compared to adults (Palin et al., 2013). Consistently, we observed a similar expression of CD69 and an increase of expression of CD25 and CD44 in neonatal CD4^+^ T cells compared to that in adult cells after *in vitro* stimulation with anti-CD3 and anti-CD28 Abs. Moreover, neonatal CD4^+^ T cells showed an increase of both proliferation ability and activation-induced cell death. These data support that CD4^+^ T cells in neonatal mice are sensitive to activation, leading to an enhanced proliferation capacity as well as an increased susceptibility to activation-induced apoptosis, partially contributing to the compromised immunity in neonates. Interestingly, tracked neonatal CD4^+^ T cells displayed similar potential in activation, proliferation, and survival to adult and tracked adult cells, which contribute to a more potent immune defense in adult mice. These findings are in contrast with the finding that CD8^+^ T cells generated at neonatal age preferentially differentiate into effectors *in vivo* (Smith et al., 2018). This could be caused by the difference between *in vitro* and *in vivo* T cells.

Neonatal CD4^+^ T cells preferentially differentiate into Th2 cells but show less potential toward Th1 cells compared to adult cells as reported (Holt, 2004). However, the differentiation potential of tracked neonatal CD4^+^ T cells is similar to that of adult and tracked adult cells. These findings indicate that while neonatal CD4^+^ T cells contribute less to the neonatal defense against pathogens, these cells contribute more to protective immunity during the adult stage. Previous studies demonstrated that neonatal CD4^+^ T cells are prone to differentiation into Treg cells after TCR stimulation, and are similarly capable to differentiate into Treg cells in presence of TGFβ (Wang et al., 2010), which is inconsistent with our polarized culture data. The controversy may due to different culture system in which they used thymic CD4^+^CD25^–^ cells whereas we used splenic CD4^+^CD25^–^CD62L^+^CD44^–^ pure naïve cells. However, tracked neonatal CD4^+^ T cells contributed to Treg differentiation and tolerance equally as adult and tracked adult cells in adults. We observed a higher differentiation potential of Th17 cell in neonatal cells compared to adult naïve CD4^+^ T cells, but exhibited the same level of potential after maturation *in vivo*. Early studies showed a decrease of Th17 cells from human neonatal cells *in vitro* and from *in vivo* EAE model compared to adult cell or mice (Hofstetter et al., 2007; de Roock et al., 2013). The differences of Treg and Th17 cell differentiation between published data and our results could be due to that we used purified naïve CD4^+^ T cells whereas others used total CD4^+^ T cells. In addition, the high frequency of activated cells or effector-like cells in neonates may contribute more to the production of Treg and Th17 cells than that in adults.

In conclusion, we used a powerful lineage tracing system to investigate the features of neonatal and tracked-neonatal CD4^+^ T cells. Our study uncovered that neonatal CD4^+^ T cells are sensitive to activation, proliferation, and apoptosis. We also showed that neonatal CD4^+^ T cells are more prone to differentiate into Th2 and Th17 cells but less for Th1 and Treg cells. In contrast, tracked neonatal CD4^+^ T cells exhibit similar potential in above-mentioned aspects as adult and tracked adult cells. Our findings emphasized the requirement of homeostatic process for neonatal CD4^+^ T cells to gain functional potential of adult cells.

## Data Availability Statement

The original contributions presented in the study are included in the article/supplementary material, further inquiries can be directed to the corresponding author/s.

## Ethics Statement

The animal study was reviewed and approved by the Institutional Animal Care and Use Committee of Xi’an Jiaotong University.

## Author Contributions

LL, XzZ, XY, YS, CS, and BZ analyzed the data and wrote the original draft. LL, XzZ, HY, HZ, XW, and JW performed the FACS analysis. HL, AJ, JZ, DZ, LS and CZ collected the samples. CS, XbZ, and BZ discussed the data analysis. CS and BZ generated the idea, designed the experiment, and wrote the manuscript. All authors agreed to be responsible for their own part of the work.

## Conflict of Interest

The authors declare that the research was conducted in the absence of any commercial or financial relationships that could be construed as a potential conflict of interest.
